# Thyroid Cancer: Toward Surgical Evolution

**DOI:** 10.3390/jcm10163582

**Published:** 2021-08-14

**Authors:** Annamaria D’Amore, Renato Patrone, Ludovico Docimo, Giovanni Conzo, Celestino Pio Lombardi

**Affiliations:** 1 Department of Gastroenterologic, Endocrine-Metabolic and Nephro-Urologic Sciences, Endocrine Surgery Division, Fondazione Policlinico Universitario A. Gemelli IRCCS, 00168 Rome, Italy; annamaria.damore@policlinicogemelli.it (A.D.); cplombardi@libero.it (C.P.L.); 2 PhD ICHT, University of Naples Federico II, 80131 Naples, Italy; 3 Department of Cardiothoracic Sciences, University of Campania “Luigi Vanvitelli”, Division of General and Oncologic Surgery, Via Pansini 5, 80131 Napoli, Italy; ludovico.docimo@unicampania.it (L.D.); giovanni.conzo@unicampania.it (G.C.)

It has been more than five years since the American Thyroid Association (ATA) and the Italian consensus on thyroid cancer was published [1,2]. It revaluated and retrospectively analyzed the results of thyroid cancer treatment and the outcomes in terms of disease-free survival and recurrence rate. Both suggested a more conservative approach for low/intermediate-risk well-differentiated thyroid cancers (DTCs), rehabilitating hemithyroidectomy as the surgical approach of choice. It is well known that DTCs represent the most common of all thyroid cancers [3]. Moreover, in recent decades, we have assisted in a continual and constant increase in the number of new diagnoses. It is quite clear that overdiagnosis and the incredible spread of neck ultrasound using thyroid nodule detection as well as fine-needle aspiration of a nodule of less than centimeter in dimension justifies this enormous number of new thyroid cancer diagnoses we have to manage daily. It is strongly evident that, usually, DTCs carry very good prognosis (10-year survival rate more than 90%) with a low rate of death, whereas, by contrast, persistent or recurrent disease is more common than the others. Furthermore, the 8th edition of the American Committee on Cancer and Union Internationale Contre le Cancer (AJCC/UICC) reviewed the DTC staging system: a larger part of tumors are now downstage to I or II because of the absence of distant metastases, even if it is smaller than 4 cm associated with microscopic extension to perithyroidal soft tissues and, in the case of lymph-node metastases, in the central and lateral compartments [4]. This new consideration leads to the identification of a small proportion of DTC patients, approximately 5–10%, who are at risk of death because of their cancer and their stage (usually III or IV) [5]. After these considerations, it is understandable that the recent guidelines push towards a new therapeutic approach to DTCs with low/intermediate risk. The previous almost paradigmatic indication of an absolute, complete, and more aggressive approach pressed physicians and endocrine surgeons in the past to prefer the typical “one-size-fits-all” mode and to suggest total thyroidectomy associated with central neck dissection, and radio iodine remnant ablation in the majority of patients. Presently, evidence suggests a new idea for surgery: a conservative approach for all DTCs with a preoperative low/intermediate risk, no evidence of lymph-node metastasis or other signs of aggressive presentation of the hilum and no other conditions that upgrade risk. The risk of thyroid cancer recurrence is common and moderately both in low and intermediate DTCs, but it is not influenced by an early surgical approach [6]. It is evaluated by international database reports and clarified in the literature that the recurrence rate between hemithyroidectomy and total thyroidectomy is similar and seems not to affect prognosis [7]. Moreover, a new surgical operation in the case of contralateral recurrence does not impact survival [8]. Even if the American and Italian guidelines are definitive towards this new concept of conservative and repeatable surgery, daily routines involving physicians and surgeons do not yet follow that line. Overdiagnosis and possible overtreatment is still prevalent in real-life cohorts in Italy, and no significant changes have been recorded in recent years. The rate of lobectomies is growing, but the procedure still represents a minority of all performed surgeries [9,10,11,12]. However, such an approach needs a solid multidisciplinary group to decide and propose strategy, to manage follow-up and establish new indications for treatment. Patients need to be well identified, all clinical features must be known and analyzed, and surgical approaches must be well explained and accepted by patients. Moreover, a wider knowledge of minimally invasive surgical techniques for thyroid diseases needs to be massively widespread, as well as the possibility for patients to refer to an endocrine center performing such surgery. Actual surgery for low and intermediate DTCs must be well fitted for every patient. In our experience, according to the guidelines and in contrast with the Italian trends, we are constantly contributing to an increased number of hemithyroidectomy performed for DTCs, which in recent months represented about 20% of all interventions for thyroid disease, compared to 5% in the last decade, with a large number of patients at follow-up stage. In this group, thyroidectomy was necessary in about 25% of cases because of evidence at final histology of extensive disease in contrast with preoperative features (histological variant, extra thyroidal disease, and/or multifocality and/or lymph-node metastases). We have a time constraint for follow-up, and a small number of cases require total thyroidectomy to produce dynamic and statistical evidence, so we are confident that a larger number of colleagues will follow this approach to implement and strengthen this guideline consensus.
